# MicroRNA-410-5p as a potential serum biomarker for the diagnosis of prostate cancer

**DOI:** 10.1186/s12935-016-0285-6

**Published:** 2016-02-19

**Authors:** Jiaqi Wang, Huamao Ye, Dandan Zhang, Yijun Hu, Xiya Yu, Long Wang, Changjing Zuo, Yongwei Yu, Guixia Xu, Shanrong Liu

**Affiliations:** Clinical Research Center, Changhai Hospital, Second Military Medical University, 168 Changhai Road, 200433 Shanghai, China; Department of Urology, Changhai Hospital, Second Military Medical University, 168 Changhai Road, 200433 Shanghai, China; Department of Anesthesiology, Changhai Hospital, Second Military Medical University, 168 Changhai Road, 200433 Shanghai, China; Departments of Nuclear Medicine, Changhai Hospital, Second Military Medical University, 168 Changhai Road, 200433 Shanghai, China; Department of Pathology, Changhai Hospital, Second Military Medical University, 168 Changhai Road, 200433 Shanghai, China

**Keywords:** miR-410-5p, Prostate cancer, Diagnosis, Circulating miRNA

## Abstract

**Background:**

Prostate cancer (PCa) remains to be a diagnostic challenge due to its variable presentation and the lack of reliable diagnosis tool. MicroRNAs (miRNAs) regulate gene in extensive range of pathophysiologic processes. Plasma miRNAs are ideal biomarkers in heart failure, diabetes and other disease. However, using circulating miRNAs as biomarkers for the diagnosis of PCa is still unknown.

**Methods:**

149 PCa patients, 57 healthy controls, and 121 non-cancer patients (benign prostatic hyperplasia and other urinary diseases) were enrolled in this study. The reverse transcription of miRNA and SYBR-Green-based double standards curve miRNA quantitative polymerase chain reactions (qPCR) were used to evaluate the dysregulated miR-410-5p. Receiver operator characteristic (ROC) curve analysis was used to evaluate the diagnostic accuracy of miR-410-5p identified as the alternative biomarker.

**Results:**

Circulating miRNA-410-5p (miR-410-5p) level was significantly higher in the PCa patients than in healthy controls or non-cancer patients. ROC curve analysis showed that plasma miR-410-5p was a specific diagnostic biomarker of PCa with an area under curve(AUC) of 0.8097 (95 % confidence interval, 0.7371–0.8823; P < 0.001).

**Conclusions:**

The serum miR-410-5p level is a potential biomarker for the diagnosis of PCa.

## Background

Prostate cancer (PCa) is the development of cancer in the prostate associated with a substantial morbidity and mortality [1, 2]. It has reported that 233,000 new cases and 29,480 deaths occurred in the year 2014 [2, 3]. Early diagnosis and treatment before tumor metastasizes is crucial for improving the patient survival [1, 4]. The 5 year survival ratio for men diagnosed while the PCa is localized is nearly 100 %. And only 28 % of the men who diagnosed with metastatic PCa survive beyond 5 years [5]. There are still to be challenges for the early diagnosis of PCa due to its variable presentation [6].

Diagnostic testing for PCa has been widely studied and searches from biomarkers [7, 8], such as the prostate-specific antigen (PSA) assay [9], to radiologic imaging, such as magnetic resonance imaging (MRI) [8, 10], and Biopsy [11]. Although widely used as a diagnosis tool, serum PSA assays are sensitive but not specific enough to detecting PCa [4, 9, 12]. Novel biomarkers with enhanced detective accuracy would greatly assist the diagnosis of PCa [13].

Recently, microRNAs (miRNAs) have been found to play crucial roles in many cancer cellular processes, such as proliferation [14, 15], differentiation [12, 16] and apoptosis [2]. MiRNAs are small, endogenous, noncoding RNAs with single-stranded RNA that regulate gene expression by combine with messenger RNAs (mRNAs) and inhibiting the translation or promoting degradation of mRNA [17, 18]. Our research found that miR-410-5p were secreted by prostate cancer cells and released into peripheral blood. We therefore sought to explore the plasma miR-410-5p as biomarkers for the diagnosis of PCa.

## Methods

### The populations of patients

Between February 2010 and July 2011, 327 patients with a high probability of PCa or those with an intermediate probability and a positive PSA enzymelinked immunosorbent assay test (ELISA) (>4 μg/L) in Shanghai Changhai Hospital were testing to confirm PCa. In accordance with the existing clinical guidelines, these patients underwent a biopsy test for prostate to confirm the prostate cancer. After the diagnosis of PCa, Gleason score was examined to evaluate the microscopic features of prostate cancer found. Serum PSA was used as a biomarker of PCa risks and treatment prognosis. Afterwards, risk stratification was evaluated according to the clinical guidelines. Briefly, high risk PCa is diagnosed if gleason score is higher than 7 [19]. Intermediate-risk PCa is confirmed gleason score is 7 and PSA is between 10–20 ug/L. Low-risk PCa is confirmed when gleason level is lower than 7 [19]. During the period, 187 of 421 patients were diagnosed to have PCa, and 149 of the 187 patients which gave informed permit were enrolled in this research. The controls included 57 healthy volunteers, 81 patients with BPH and 40 patients with other urinary diseases. Moreover, 44 of 85 high-risk PCa patients were treated with surgery and others were treated with radiation therapy and chemotherapy. Approval was obtained from the ethical management committees of the Second Military Medical University. All participants gave written informed permit before sampling in the study.

### Serum sampling and total RNA isolation

At presentation, peripheral blood samples for miR-410-5p detection were collected in coagulation-promoting tubes [20] (BD, New York, USA) and processed within 1 h of collection. After centrifugation (4 °C at 3000*g* for 5 min), supernatant was transferred to RNase -free tubes and stored at −80 °C.

Total RNA was isolated from serum using a Trizol LS isolation kit (Thermo Fisher, Massachusetts, USA) according to the manufacturer’s instructions [21]. Briefly, 300 μL of human serum was used on the total RNA isolation. Each product of isolation was eluted in 50 μL of RNAse-free water. RNA quantification of human plasma samples was done by Spectrophotometric. The absorbance on 230, 260, 280 and 330 nm was detected to evaluate the severity of undetermined contaminants in isolation products. Thus, all RNA samples were analyzed for U6 expression, a stable endogenous reference non-coding RNA [22], to assess the approximate yield of RNA islation and to ensure that proportionate amounts of serum were used in each reaction of reverse transcription and qPCR.

### Quantification of miR-410-5p expression

The miRNAs reverse-transcription were performed using the Miscript RT kit [23] (#218161,QIAGEN, Germany), and the products were re-package into two tubes. QPCR with SYBR Green (#218073 and #218300, QIAGEN, Dusseldorf, Germany) were performed on RNA from serum samples of 57 randomly selected PCa patients and 14 healthy controls. QPCR steps were performed on Rotorgene 6000 Real-Time PCR System (Corbett, Sydney, Australia); the results were expressed as Ct values and normalized to calculate the average Ct of each sample (ΔCt). The relative expression of miR-410-5p was calculated using comparative Ct method [24] (2-ΔΔCt). Amplification of U6 was done with primers: 5′-CTCGCTTCGGCAGCACA-3′; 5′-AACGCTTCACGAATTTGCGT-3′ as internal control [25]. All tests were run in triplicate to minimize the experimental error.

Single miRNA expression was determined using SYBR Green-based miRNA qRT-PCR (#218073 and #218300, QIAGEN, Dusseldorf, Germany) according to the manufacturer’s instructions. The qRT-PCR was performed on 327 samples mentioned before. Briefly, the 15 μL RT reaction master mix was created with 5 μL of total RNA sample which was isolated as described above. Reverse transcription and qPCR was carried out using the Rotorgene 6000 Real-Time PCR System (Corbett, Sydney, Australia) on 20 μL of PCR master mix containing 10 μL of SYBR-Green QPCR Master Mix, 1 μL of primer, 1 μL of RT products and 8 μL of RNase free water. The qPCR reactions were performed in triplicate. Due to the lack of generally accepted standards, double standard curve was made for endogenous control and quantification. All reactions were run in triplicate. QPCR products of miR-410-5p and U6 was recombined into pMD18T as standards [26].

For comparison test, qPCR was performed on 73 RNA samples from the cohort provided above (Including 34 randomly selected PCa patients, 25 BPH patients and 14 healthy controls). The relative expression of miR-410-5p, miR-1228 and let-7c was calculated using comparative Ct method. Amplification of let-7c was performed as a positive control according to the following Ref. [27, 28] and amplification of miR-1228 was performed as a negative control [29].

### Statistical analysis of miR-410-5p

Data characterized by the normal classification were expressed as the average and standard deviation. miR-410-5p content is widely presented using the double standard curve method. Recombinant vector pMD18T-miR-410-5p and pMD18T-U6 were proliferated, extracted with DH5a and accurately quantified as the standards [26]. The Ct values of samples were compared with standards to calculate the copy numbers [30]. A Chi squared test,independent samples *t* test or one-way analysis of variance (ANOVA) was done when appropriate. If significant differences were found, a Bonferroni post hoc test was done to determine which teams differed significantly according to equal variance criterion. After that, the receiver-operator characteristic curve (ROC) analysis was performed with plasma miR-410-5p distinguishing between PCa patients and non-cancer controls or different stage PCa patients. The value of area under ROC curve (AUC) was estimated to criticize the diagnostic accuracy of miR-410-5p. All analyses were performed using Microsoft Excel 2013 by two-sided. For all analyses, p values <0.05 were considered statistically significant [31].

## Results

### miR-410-5p contents in the plasma of PCa patients

**Fig. 1:**
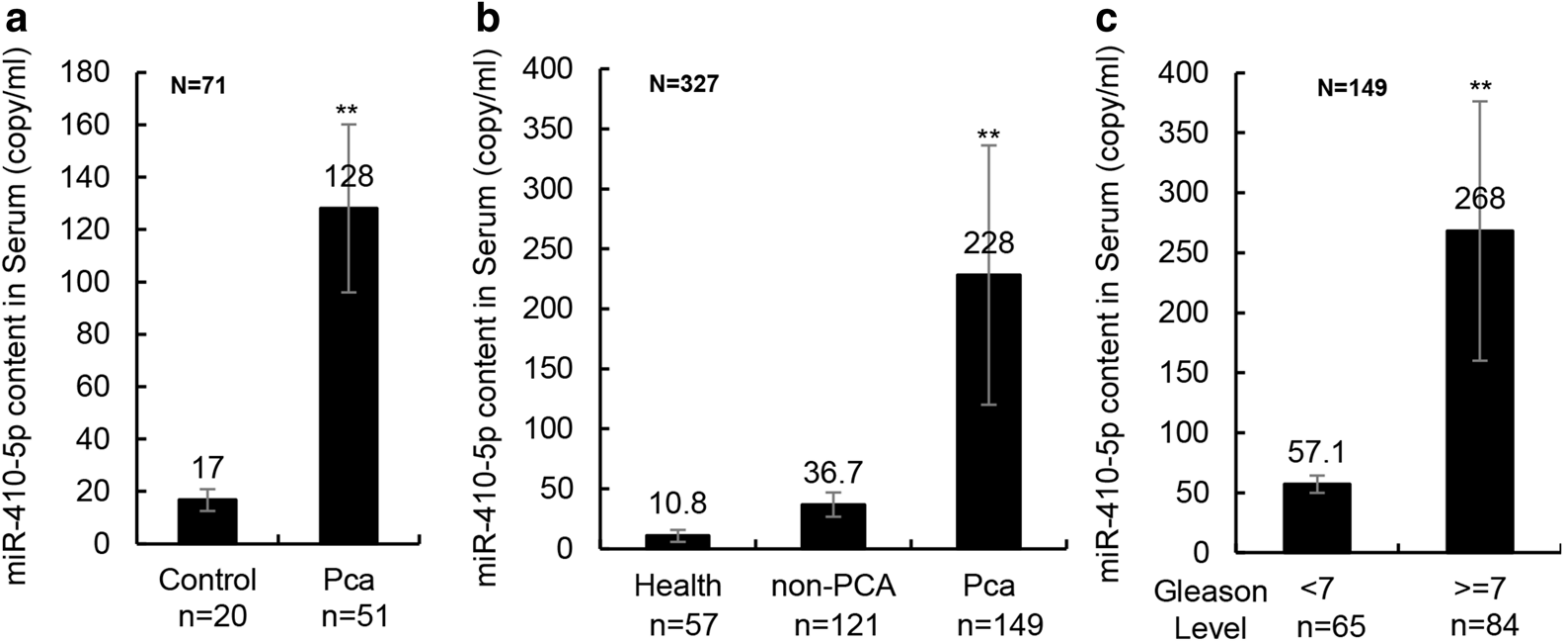
Serum miR-410-5p levels in different groups. **a** The relative serum miR-410-5p levels in 20 healthy controls and 51 PCa patients. The value of miR-410-5p was provided above the histogram. **, P < 0.01. **b** The Serum miR-410-5p level in 57 healthy volunteers, 121 non-PCA controls (include 81 patients with BPH and 40 patients with other urinary diseases) and 149 PCA patients. The value of miR-410-5p was provided above the histogram. **, P < 0.01. **c** The Serum miR-410 levels in 65 low-risk PCa patients (Gleason level <7) and 84 high-intermediate-risk PCa (Gleason level >=7). The value of miR-410-5p was provided above the histogram. **, P < 0.01

Plasma miR-410-5p were identified as the biomarker in 57 PCa patients from 14 healthy controls (Fig. 1a). The elevation of miR-410-5p was validated using double standard curve qRT-PCR in 149 PCa patients and 178 non-cancer controls (Fig. 1b). The basic clinical characteristics of PCa patients and non-cancer patients are shown in the Table 1. It showed that there were no remarkable differences in age between the PCa and non-cancer groups. These data have been normalized by double standard curves, a widely used normalized method for qPCR that was also confirmed in our other research. Moreover, we compared the difference in miR-410-5p content between 85 high-intermediate risk PCa patients and 64 low-risk PCa patients and obtained the same differences in miR-410-5p expression (Fig. 1c), which further supports that miR-410-5p is a stable biomarker for PCa.

**Table 1:** Clinical characteristics of prostate cancer (PCA) patients and non-PCA patients

Characteristics	PCa (n = 149)	Non-PCa (n = 178)
Age	73.55 ± 7.36	73.25 ± 6.13
PSA	24.62 ± 24.56	1.78 ± 0.88
Risk stratification		
Low risk	57	–
Intermediate risk	51	–
High risk	41	–
Recurrence		
Biochemical	35	–
Metastasis	14	–
Treatments		
Surgery	47	3
Radiotherapy	27	0
Hormonotherapy	18	0

**Fig. 2:**
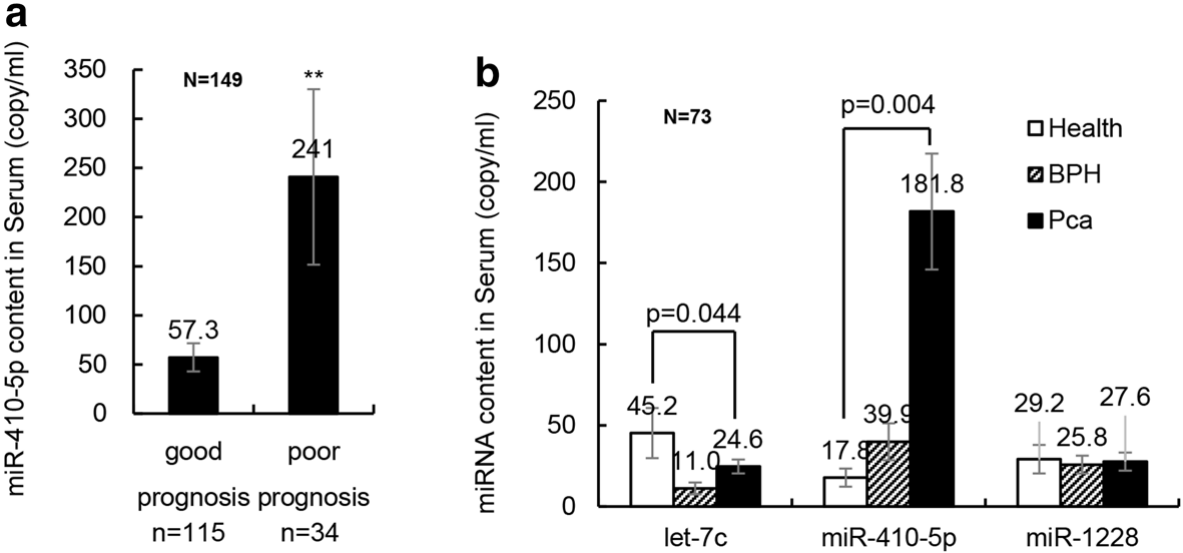
Serum miR-410-5p levels in PCa patients and comparison test with miR-1228 and let-7c. **a** The relative serum miR-410-5p level in 115 good prognosis patient (no recurrence) and 34 PCa patients with poor prognosis. The expression levels of miR-410-5p was normalized by U6. The value of miR-410-5p was provided above the histogram. **, P < 0.01. **b** The relative serum level of let-7c, miR-410-5p, and miR1228 in 73 samples (Including 34 randomly selected PCa patients, 25 BPH patients and 14 healthy controls). The expression levels were normalized by U6. The value of miRNAs level and p value were provided above the histogram

The plasma miR-410-5p level was increased in the PCa group compared to both the non-cancer group and healthy controls (Fig. 1a). However, plasma miR-410-5p was also higher in the serums of PCa patients without recurrence than in the serums of PCa patients with poor prognosis (Fig. 2a), indicating that miR-410-5p may be a stable biomarker to evaluate the prognosis of PCa. To confirm that the assay is reproducible, all tests were repeated twice. It must be confirmed that no remarkable difference in the content of miR-410-5p was found between three tests of one sample. We also compared the plasma miR-410-5p level between high-intermediate risk PCa and low-risk PCa and found that the miR-410-5p level was significantly higher in the low-risk patients compared to high-intermediate-risk PCa patients (Fig. 1c).

It’s also found approximately distinctions between PCa patients and healthy controls in plasma miR-410-5p levels and let-7c levels (Fig. 2b). Let-7c was known as a potential biomarker for PCa diagnosis [27, 28] and was used as positive control in this study. QPCR of miR-1228 was performed as a negative control for its steady expression in the blood [29].

### Diagnostic accuracy of plasma miR-410-5p for PCa

**Fig. 3:**
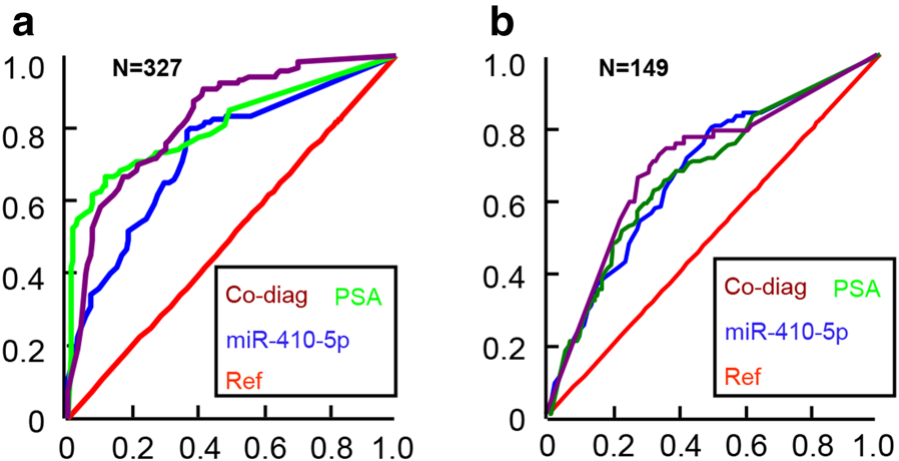
ROC curve test of serum miR-410-5p. **a** ROC curve of miR-410-5p, PSA and both for all patients with PCa versus all controls in 327 samples. The optimum cutoff for miR-410-5p expression and PSA was 21.4 copy/ml and 4 pg/ml, respectively for diagnosis for PCa. **b** ROC curve of miR-410-5p, PSA and both for low-risk PCa (Gleason score <7) versus high-intermediate-risk PCa (Gleason score >=7) in 149 PCa patients. The optimum cutoff for miR-410-5p expression and PSA was 64.1 copy/ml and 13.12 ng/L, respectively

The ROC curve analysis was performed to evaluate the diagnostic accuracy of serum miR-410-5p. When a comparison was made between the PCa group compared to the non-cancer control group, the AUC value was 0.8097 (95 % confidence interval, 0.7371–0.8823; P < 0.001) (Fig. 3a). When the comparison was made between the high-intermediate risk PCa and low-risk PCa group, the AUC value was 0.7125 (95 % confidence interval, 0.6292–0.7958; P = 0.002). Using 21.4 ng/L as a threshold for the serum miR-410-5p level, the specificity and sensitivity of plasma miR-410-5p for the diagnosis of PCa in patients reporting PSA >4 ng/L (Fig. 3b). In addition, co-diagnosis by plasma miR-410-5p and PSA distinguished PCa cases from healthy controls plus non-PCa cases had an AUC value of 0.8274 (95 % confidence interval, 0.7029–0.9519; P < 0.001) (Fig. 3a).

## Discussion

PCa has a nonspecific clinical biomarker presentation and is hard to diagnose [4, 6]. Although great progress has been found in the detection and exclusion of PCa with the advent of PSA assay [1], radiologic imaging [10] and biopsy [11], there is still a superior need for specific and reliable biomarker for the detection early diagnostic testing of PCa. In our study, we confirmed serum miR-410-5p as a potential biomarker for PCa [13]. Ideally, a biomarker should be repeatable and have a high specificity and sensitivity for the diagnosis of a pathognomonic disease [32]. miRNAs are suitable potential biomarkers because of their fulfilling many of these criteria [33–36]. Furthermore, miRNAs are present in human peripheral blood in a greatly stable form that is protected from RNase activity and remain stable even in harsh conditions [17, 24, 37]. The stability, lower structure complexity, and lack of modifications make circulating miRNAs to be ideal diagnostic biomarker candidates [38]. The high specificity and sensitivity of miRNA detection using reverse transcription and qPCR may create accurate cut-off values for diagnosis. Until now, the function of serum miR-410-5p in PCa has not been reported. As confirmed by the miR-410-5p in this study, the application of miRNAs as minimally stable and sensitive biomarkers would result in great breakthroughs for the diagnosis of common disease [39, 40].

PSA assay and biopsy test is widely used in the clinical PCa diagnosis. The sensitivity of PSA assay alone was 82.4 %, whereas the combination of PSA assay and biopsy test increased the sensitivity to 87.1 % [41]. However, the specificity of PSA assay is not enough to make a definite diagnosis on PCa, and biopsy is a traumatic test on prostate and cannot improve the quality-adjusted life-year (QALY) [42]. Plasma miR-410-5p might be an appropriate alternative. In addition, using plasma miR-410-5p was not a traumatic test and the co-diagnosis improves the specificity of PSA.

We have demonstrated that the plasma miR-410-5p level was not affected by non-cancer conditions. Plasma miR-410-5p could distinguish PCa cases from healthy controls or non-cancer cases with an AUC value of 0.8097 or 0.7652, which indicates that serum miR-410-5p could to be a potential biomarker to diagnose PCa. Furthermore, our recent research confirmed that the expression of miR-410-5p was 7.5-fold higher in the peripheral blood dendritic cells (DCs) of PCa patients compared to non-cancer controls. In this study, the serum miR-410-5p level in 26 patients with chronic prostatitis and 14 with an acute urinary tract infection was similar to the healthy controls. This result may suggest the function of miR-410-5p and give an explanation for the expression and secrete of miR-410-5p.

Our study was subject to several limitations. First, for clinical study, 327 patients (include 149 PCa patients) were relatively small in scale. And the results will require further replication in independent studies of PCa. Second, it would to be necessary to study the function of miR-410-5p in both PCa and other-disease patients. Third, it would be helpful to research whether combining the values of serum miR-410-5p content and PSA assay would greatly enhance the sensitivity and specificity for plasma miR-410-5p. Further studies are needed to resolve this tissue specificity. However, the PSA assay was a high-sensitivity and low-specific diagnostic method because it is also positive in the patients with prostatitis, benign prostatic hyperplasia, and other prostate diseases [42]. The specificity of miR-410-5p in diagnosing PCa was better than PSA assay in this study. Forth, further study is required to determine the additional benefit of miR-410-5p in staging and prognostic of prostate cancer. Fourth, the function of plasma miR-410-5p is still unclear. It is commonly speculated that circulating miRNAs play key role in maintaining the homeostatic state of the circulatory system [33, 34]. But our research revealed that miR-410-5p assembling in DCs. Whether plasma miR-410-5p can trigger some pathogenic effects in dysfunction of DCs in PCa patients remains unclear. Finally, the pathogenic mechanism of miR-410-5p levels and the relationship with PCa is unclear. Our prior studies have confirmed that the release of miR-410-5p from prostate cancer cells may be cause of the immunologic escaping in PCa.

## Conclusions

In conclusion, we confirmed that elevated serum miR-410-5p level is a potential biomarker for the diagnosis of PCa. Our results provide a basement for future efforts to develop serum miR-410-5p-based assays to diagnose PCa.
